# Economic costs analysis of uncomplicated malaria case management in the Peruvian Amazon

**DOI:** 10.1186/s12936-020-03233-5

**Published:** 2020-04-21

**Authors:** Diamantina Moreno-Gutierrez, Angel Rosas-Aguirre, Alejandro Llanos-Cuentas, Joke Bilcke, José Luis Barboza, Marie-Pierre Hayette, Juan Contreras-Mancilla, Kristhian Aguirre, Dionicia Gamboa, Hugo Rodriguez, Niko Speybroeck, Philippe Beutels

**Affiliations:** 1 Facultad de Medicina Humana, Universidad Nacional de la Amazonía Peruana, Iquitos, Loreto 160 Peru; 2 Research Institute of Health and Society (IRSS), Université Catholique de Louvain, 1200 Brussels, Belgium; 3 Centre for Health Economics Research and Modelling Infectious Diseases, Vaccine and Infectious Disease Institute, University of Antwerp, 2000 Antwerp, Belgium; 4 Fund for Scientific Research FNRS, 1000 Brussels, Belgium; 5 Instituto de Medicina Tropical “Alexander von Humboldt”, Universidad Peruana Cayetano Heredia, Lima, 31 Peru; 6 Facultad de Salud Pública y Administración, Universidad Peruana Cayetano Heredia, Lima, 31 Peru; 7 Department of Clinical Microbiology, Center for Interdisciplinary Research on Medicines (CIRM), University Hospital of Liège, 4000 Liège, Belgium; 8 Laboratorios de Investigación y Desarrollo, Facultad de Ciencias y Filosofia, Universidad Peruana Cayetano Heredia, Lima, 31 Peru; 9 Departamento de Ciencias Celulares y Moleculares, Facultad de Ciencias y Filosofia, Universidad Peruana Cayetano Heredia, Lima, 31 Peru

**Keywords:** Economic, Cost, Malaria, Management, Health care-seeking behaviour, Peru

## Abstract

**Background:**

Case management is one of the principal strategies for malaria control. This study aimed to estimate the economic costs of uncomplicated malaria case management and explore the influence of health-seeking behaviours on those costs.

**Methods:**

A knowledge, attitudes and practices (KAP) survey was applied to 680 households of fifteen communities in Mazan-Loreto in March 2017, then a socio-economic survey was conducted in September 2017 among 161 individuals with confirmed uncomplicated malaria in the past 3 months. Total costs per episode were estimated from both provider (Ministry of Health, MoH) and patient perspectives. Direct costs were estimated using a standard costing estimation procedure, while the indirect costs considered the loss of incomes among patients, substitute labourers and companions due to illness in terms of the monthly minimum wage. Sensitivity analysis evaluated the uncertainty of the average cost per episode.

**Results:**

The KAP survey showed that most individuals (79.3%) that had malaria went to a health facility for a diagnosis and treatment, 2.7% received those services from community health workers, and 8% went to a drugstore or were self-treated at home. The average total cost per episode in the Mazan district was US$ 161. The cost from the provider’s perspective was US$ 30.85 per episode while from the patient’s perspective the estimated cost was US$ 131 per episode. The average costs per *Plasmodium falciparum* episode (US$ 180) were higher than those per *Plasmodium vivax* episode (US$ 156) due to longer time lost from work by patients with *P. falciparum* infections (22.2 days) than by patients with *P. vivax* infections (17.0 days). The delayed malaria diagnosis (after 48 h of the onset of symptoms) was associated with the time lost from work due to illness (adjusted mean ratio 1.8; 95% CI 1.3, 2.6). The average cost per malaria episode was most sensitive to the uncertainty around the lost productivity cost due to malaria.

**Conclusions:**

Despite the provision of free malaria case management by MoH, there is delay in seeking care and the costs of uncomplicated malaria are mainly borne by the families. These costs are not well perceived by the society and the substantial financial impact of the disease can be frequently undervalued in public policy planning.

## Background

The good economic performance together with targeted social spending and enhanced anti-poverty programs led Peru to have significant success in improving health outcomes during the Countdown to the 2015 Millennium Development Goals (MDGs) [1]. Currently, the 2030 Sustainable Development Goals (SDGs) “*to ensure healthy lives and promote wellbeing for all at all ages*” [2] challenge the health sector, not least because Peruvian health expenditure are among the lowest in the Americas (about 5% of Gross Domestic Product-GDP) [3], substantial health inequities persist [4], and communicable diseases such as malaria resurge [5, 6].

Malaria remains an important public health problem in the country despite several decades of intense control efforts [7]. Peru had the third highest increase in malaria incidence (after Venezuela and Nicaragua) since 2010 in the Americas, [8], having reported about 72% more malaria cases in 2017 (54,309 cases) compared with 2010 (31,545 cases) [9]. The Amazon Region, mainly the department of Loreto, is commonly affected by malaria due to both *Plasmodium vivax* and *Plasmodium falciparum* (*P. vivax*/*P. falciparum* ratio: 4/1), accounting for more than 95% of Peru’s malaria cases in 2017 (52,280 cases) [9]. The complex association between poverty and malaria is well known, which likely operates in both directions in the poorest districts of Loreto: poor households are more exposed to infectious mosquitoes due to occupational hazards (i.e. subsistence farming and hunting) and less able to afford prevention, and the higher burden of malaria may push these same households deeper into poverty (due to high productivity losses following multiple episodes throughout a year) [10, 11]. Indeed, a former economic evaluation conducted in 1998 from the perspective of society found that malaria costs were mainly borne by the families [11].

In the last two decades, the Peruvian Ministry of Health (MoH) has implemented strategies to improve the population’s access to healthcare, especially for people not covered by the contributory social health insurance system (EsSalud), the Armed Forces (FFAA), National Police (PNP), or the private sector [12]. Thus, the Integrated Health Insurance scheme (IHIS), launched in 2009 by MoH, allowed for the expansion of former benefit packages beyond maternal and child health, and the increase of healthcare coverage to the poor and vulnerable populations of all ages [13–15]. Currently, the IHIS and EsSALUD cover about 65% and 17% of the total population in Loreto, respectively [16]; however, in poor Amazonian districts that consist mostly of rural communities, the IHIS is often the only possibility to provide health services to the population. The IHIS has reinforced the main pillar interventions for malaria control in Loreto, such as malaria surveillance and case identification through passive case detection (PCD, i.e. malaria detection at health facility or by community health workers (CHW) using standard diagnosis among symptomatic individuals seeking healthcare) or active case detection (ACD, i.e. malaria detection in the community through household visits) [17, 18], and the effective, free, and timely management of confirmed malaria infections. In the past, those interventions were mainly supported by funding specifically assigned to the National Malaria Control Program (NMCP) at MoH [11].

From an economic perspective, it was described that the healthcare-seeking behaviours among individuals with malaria-like symptoms can significantly affect the direct and indirect costs associated with malaria detection and management [19]. In areas like Loreto, early and appropriate healthcare-seeking behaviours can save direct costs by halting transmission from confirmed malaria infections, and by avoiding additional costs associated with complications of such infections [11, 20]. Conversely, delayed behaviours, self-medication, and seeking healthcare from traditional healers can increase the latter direct medical costs, impacting household budgets due to the lost wages and transportation costs during the care-seeking process and the illness period [20]. This study aimed to estimate the economic costs associated with the PCD and management of uncomplicated malaria episodes in riverine communities in the Peruvian Amazon from both the provider’s and patient’s perspectives, and explore whether healthcare-seeking behaviours significantly influence those costs.

## Methods

### Study design and study area

An epidemiological and knowledge, attitude and practice (KAP) survey, followed by a socio-economic survey were conducted in Mazan district, in the northeastern Peruvian Amazon Department of Loreto. Mazan, with a population of about 13,900 inhabitants living in 70 riverine communities, is considered one of the districts with the highest risk for malaria transmission in Peru [9]. In 2017, its annual parasite index (API) reached 96.4 cases per 1000 inhabitants. Malaria is predominantly caused by *P. vivax* (74%), and less by *P. falciparum* (26%), while its main vector is *Anopheles (Nyssorhynchus) darlingi* in Peru [21]. The capital and largest village in Mazan, i.e. Mazan town (MT), is located at the confluence of the Mazan and Napo Rivers (3.503° S, 73.094° W), at about 55–60 km (1 hour by speedboat) from Iquitos city (capital of Loreto).

Malaria diagnosis and treatment in healthcare-seeking individuals with malaria-like symptoms are provided free of charge by the MoH in all Peruvian malaria endemic areas. In Mazan, a health centre in MT and health posts located in six riverine communities are responsible for these activities [22]. With a laboratory service available 6 days a week and two microscopists with accredited competency in species identification according to World Health Organization (WHO) standards [23], the health centre in MT is the only health facility in Mazan that provides microscopic diagnosis of malaria and immediate anti-malarial treatment to microscopically-confirmed infected individuals. Unless rapid diagnostic tests (RDTs) are available, health posts need to collect blood smear samples from symptomatic individuals, send them to the health centre, and wait for the parasitological confirmation to initiate anti-malarial treatment. In communities with lack of health facilities, these activities are performed by CHWs, who are regularly trained and supervised by the health facilities. The available RDT during the study period was the SD Bioline Malaria Ag Pf/Pf/Pv (Standard Diagnostics, South Korea), which detects three malaria antigens in human whole blood: the histidine-rich protein II (HRP-II) antigen of *P. falciparum*, the *Plasmodium* lactate dehydrogenase (pLDH) of *P. falciparum* and the pLDH of *P. vivax* [24].

According to national guidelines, *P. vivax* malaria episodes without criteria of severity and hospital admission (i.e. uncomplicated malaria) are treated immediately with chloroquine (CQ) for 3 days (10 mg/kg on days 1 and 2, and 5 mg/kg on day 3) plus primaquine (PQ) (0.5 mg/kg/day) for 7 days; while the first-line treatment for uncomplicated *P. falciparum* malaria consists of artesunate (AS) for 3 days (4 mg/kg/day), mefloquine (MQ) for 2 days (12.5 mg/kg/day) and PQ (0.75 mg/kg) in single dose. Uncomplicated *P. falciparum*–*P. vivax* coinfections receive AS and MQ at the same doses than those for uncomplicated *P. falciparum* infections, but together with an extended period of PQ (0.5 mg/kg/day) for 7 days [25]. In vivo clinical studies have found that both CQ and the combination MQ-AS remain efficacious for the treatment of respectively uncomplicated *P. vivax* and *P. falciparum* infections in the Peruvian Amazon [26, 27].

### Study population

**Fig. 1:**
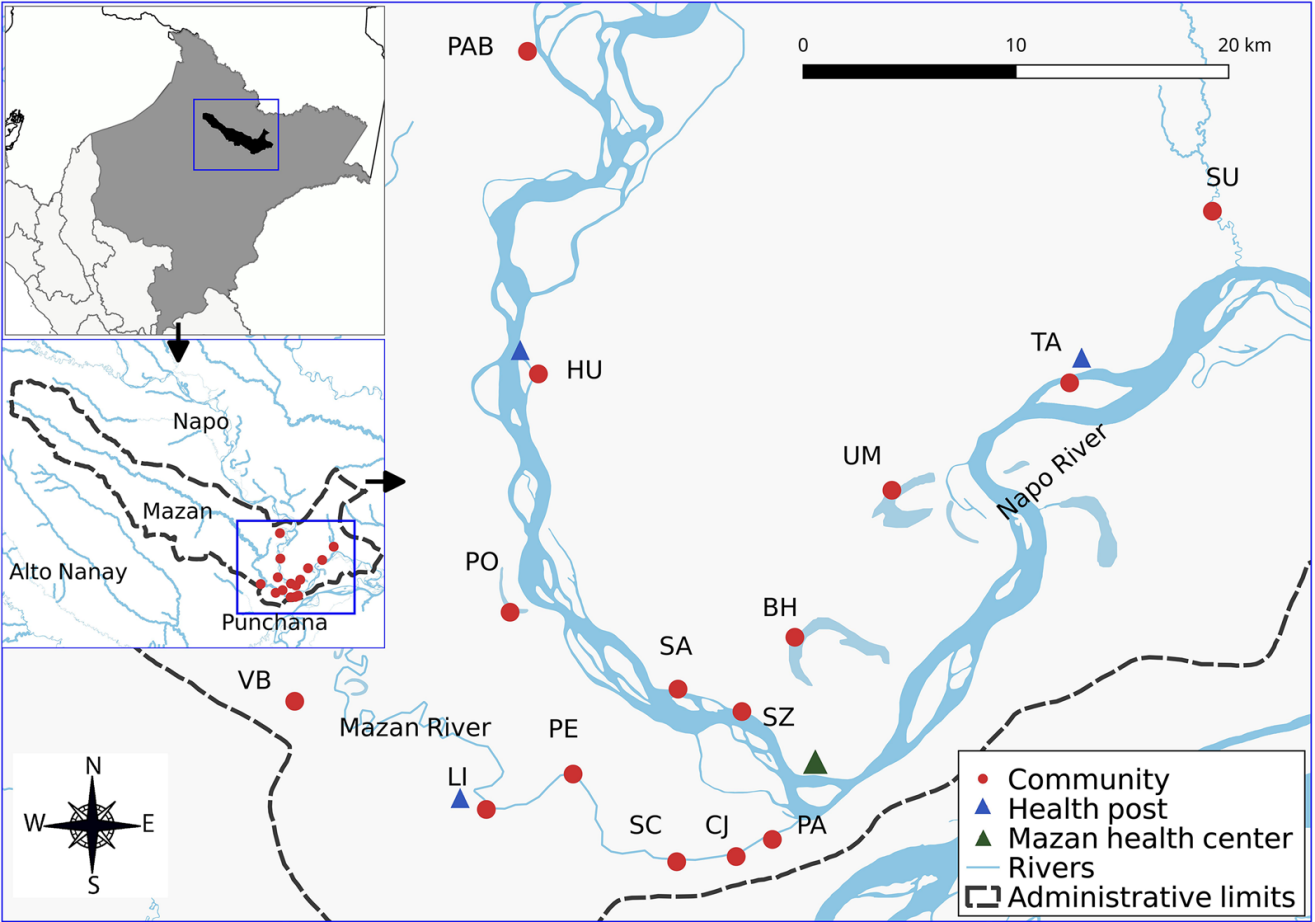
Study communities in Mazan Loreto, Peru. *BH* Bello Horizonte, *CJ* Catorce de Julio, *HU* Huaman-Urco, *LI* Libertad, *PAB* Puerto Abeja, *PA* Puerto Alegre, *PE* Primero de Enero, *PO* Puerto Obrero, *SA* Salvador, *SZ* San Antonio de Zambrano, *SC* Santa Cruz, *SU* Sucusari, *TA* Tamanco, *UM* Urco Miraño, *VB* Visto Bueno

The study population were all households from fifteen communities in Mazan district (Fig. 1). These communities were chosen among the total 70 communities in Mazan, because they accounted for 80% of reported cases in the district in the period 2016–2017. The Table 1 provides information of study communities about the availability of health facilities, distance in minutes to the health centre, and population. Inhabitants live mainly in open or semi-open wooden houses built on stilts. Subsistence farming is the main economic activity, followed by seasonal logging. Mestizos comprised the majority of the study population [28]; Yagua, Maijuna and Kichwa ethnic groups were present in three communities [29].

**Table 1:** Health services availability in communities, time to health centre and knowledge, attitudes and practices (KAP) survey

River basin	Community	Availability		Time to the health centre	KAP survey		
		Health post^a^	Community health worker	Minutes^b^	Inhabitant	Household	
					n	n	%
Mazan	Primero de Enero	No	Yes	135	133	25	3.7
	Visto Bueno	No	Yes	275	79	15	2.2
	14 de Julio	No	Yes	30	136	23	3.4
	Santa Cruz	No	Yes	45	439	73	10.7
	Libertad	Yes	Yes	155	374	64	9.4
	Puerto Alegre	No	Yes	15	353	57	8.4
Subtotal Mazan					1514	257	37.8
Napo	Huaman Urco	Yes	Yes	195	502	85	12.5
	San Antonio de Zambrano	No	Yes	30	124	24	3.5
	Urco Miraño^c^	No	No	225	371	54	7.9
	Bello Horizonte	No	Yes	80	285	47	6.9
	Puerto Abeja^c^	No	Yes	315	109	19	2.8
	Tamanco	Yes	No	180	228	49	7.2
	Puerto Obrero	No	No	105	109	21	3.1
	Salvador	No	Yes	45	456	91	13.4
	Sucusari^c^	No	Yes	270	179	33	4.9
Subtotal Napo					2363	423	62.2
Total					3877	680	100

^a^Health post without capacity for malaria diagnosis by light microscopy

^b^Time from community to the health centre in Mazan town using a 13 horsepower motor on a local boat

^c^Includes ethnic groups (Urco Miraño: Yagua; Puerto Abeja: Kichwa; Sucusari: Maijuna)

### Data collection

The study field team, composed of two trained health technicians and two nurses, traveled to selected communities in motorized boats and collected data over a few days. An epidemiological and KAP survey was conducted in all available households in March 2017. Prior informed consent, household members were censused, the house location geo-referenced, and the socio-demographic characteristics were collected (e.g. household size, predominant construction materials in house, ownership of bed nets, and availability of essential services such as water for domestic use, sanitation facility and electricity). A KAP survey questionnaire was applied to the household’s head or the designate to gather information on family’s attitudes, understanding of malaria transmission, recognition of signs and symptoms, perceptions of cause, care-seeking patterns, preventive measures and practices. Questionnaires were programmed using the Open Data Kit (ODK) application on mobile devices without a network connection.

A second survey (socio-economic survey) was conducted in September 2017 to obtain detailed information about the household’s care-seeking process and the cost of illness. The survey questionnaire was applied to a sub-population of households, of which at least one member had been diagnosed with uncomplicated malaria in the previous 3 months before the survey (June–August 2017). Households were visited up to three times in a period of three days to maximize study participation. When recurrent episodes were recorded for an individual in the past 3 months, only those recorded at intervals of more than 28 days were considered as independent episodes. Besides the registration of socio-demographic data and clinical features of malaria episodes on mobile devices, participants or responsible relatives (in case of minors) were also asked to complete a semi-structured questionnaire on treatment-seeking. The latter contained questions on all the places they visited to seek care/treatment, whether they had blood tests and the type of these tests, the time between the onset of symptoms and the confirmation of malaria infection, and the type of treatment they received. The questionnaire also allowed obtaining specific data for the estimation of direct and indirect household costs associated with malaria episodes. These included costs of transport during the care-seeking process, time lost from work due to illness for patients, their carers/companions (i.e. individuals who care informally for the patient at home, and/or accompany him/her during the healthcare-seeking) and substitute labourers (i.e. economically active individuals who substitute malaria patients at work).

### Data analysis

The analysis was performed using STATA 12.1 software (Statacorp, College Station, TX, USA) and R v.2.15 software (R Development Core Team, R Foundation for Statistical Computing, Vienna, Austria). Household characteristics from KAP survey, and individual socio-demographic data from the socio-economic survey were described using absolute and relative frequencies.

Univariate and multivariate generalized linear models with gamma distribution were used to test the association between the time lost due to illness and the time to malaria diagnosis (i.e. time from onset of symptoms to malaria confirmation by diagnostic test) in economically active patients (≥ 15 years) [30]. Adjusted mean ratios (Adj.MR) and the corresponding 95% confidence intervals (95% CI) measured how much more time patients lost due to illness, considering the *Plasmodium* species that caused the malaria episode, the diagnostic test that confirmed malaria, gender, education level and the time to the health centre in MT as potential confounders. All statistical analysis considered a p value < 0.05 as statistically significant.

### Cost estimates

Costs associated with uncomplicated malaria episodes in Mazan were estimated for the year 2017 from both the provider and patient perspectives. These estimates drew on the number of malaria episodes registered by the PCD’s surveillance in the district, as well as base case parameters (i.e. cost parameters or unit costs). Table 2 shows that the socio-economic survey provided data of cost parameters for estimating costs from the patient perspective; while costing databases from the MoH were required to estimate costs from the provider perspective since malaria detection and case-management is provided free-of-charge at health facilities [31]. Units costs in Peruvian Nuevos Soles (PEN) were converted into United States Dollars (US$) using the average exchange rate for 2017 (one US$ = 3.3 PEN) [33].

**Table 2:** Parameters, base case estimates and uncertainty distributions for sensitivity analysis

Parameter	Distribution	Base case (mean, proportion)	Standard error (SE)	Source
Malaria in Mazan in 2017, episodes	–	1344	–	EDB
Malaria episodes caused by Pf, proportion	–	0.23	–	EDB
Economically active (EA) Pv patient, probability	Beta	0.56	0.04	SES
Economically active (EA) Pf patient, probability	Beta	0.69	0.06	SES
Economically active (EA) companion of a Pv patient, probability	Beta	0.83	0.03	SES
Economically active (EA) companion of a Pf patient, probability	Beta	0.73	0.06	SES
Confirmed malaria by LM or RDT among symptomatic individuals, proportion	–	0.16	–	EDB
Healthcare-seeking with CHW (and not health facilities), proportion	Beta	0.17	0.03	SES
Utilization of RDT (instead of LM) at health facilities for malaria confirmation, proportion	Beta	0.21	0.03	SES
Cost of complete Pv malaria treatment, unit cost, US$	Gamma	1.32	0.38	CDB
Cost of complete Pf malaria treatment, unit cost, US$	Gamma	8.76	2.76	CDB
Cost of consultation, staff-related unit cost, US$	Gamma	1.81	0.05	CDB
Cost of consultation, non staff-related unit cost, US$	–	0.77	–	CDB
Cost of diagnosis using LM, staff-related unit cost, US$	Gamma	0.50	0.01	CDB
Cost of diagnosis using LM, non staff-related unit cost, US$	–	0.21	–	CDB
Cost of diagnosis using RDT, staff-related unit cost, US$	Gamma	0.50	0.01	CDB
Cost of diagnosis using RDT, non staff-related unit cost, US$	–	0.40	–	CDB
Cost of diagnosis using RDT, test-related unit cost, US$	Gamma	1.07	0.33	CDB
Cost of transport Pv patient, unit cost, US$	Gamma	2.36	0.35	SES
Cost of transport companion of Pv patient, unit cost, US$	Gamma	1.94	0.31	SES
Cost of transport Pf patient, unit cost, US$	Gamma	2.78	1.17	SES
Cost of transport companion of Pf patient, unit cost, US$	Gamma	0.64	0.23	SES
Time lost due to illness in an EA Pv patient, person-days	Gamma	10.21	0.99	SES
Time lost due to illness in an EA Pf patient, person-days	Gamma	11.80	1.48	SES
Time lost in EA companions of a Pv patient, person-days	Gamma	7.73	0.50	SES
Time lost in EA companions of a Pf patient, person-days	Gamma	6.85	0.90	SES

Pv, *P. vivax*; Pf, *P. falciparum*; LM, light Microscopy; RDT, rapid diagnostic test; CHW, community health worker; CDB, cost database; EDB epidemiologic database; SES, socio-economic survey

#### Provider perspective, direct costs

Direct costs from the provider perspective are costs related to the malaria detection (i.e. initial consultations), diagnosis confirmation (using LM or RDTs), appropriate treatment (i.e. antipyretic drugs, anti-malarial drugs) and follow-up (consultation and LM after treatment). The unit costs for care services provided by voluntary CHW were estimated using the standard shadow price approximation based on the 2017 national minimum wage of US$ 257.6 per month. Moreover, the unit cost’s components (i.e. staff costs, drug/supply costs, or other non-staff costs) that most contributed to the unit cost‘s variability of care services provided by either health facilities or CHW were identified. As expected, the different salary levels between health staff, resulting in different costs of their time dedicated to a given care service, contributed most to the variability of unit costs. A malaria positivity rate of 15.5% (base case parameter in 2017) among symptomatic individuals seeking for care was assumed to estimate the number of initial consultations that allow for the identification of malaria cases. It was assumed that a symptomatic individual seeking for care had only one initial consultation. The standard treatment of malaria episodes by species [25] was costed considering that patients ≥ 15 years old had an average of 60 Kilograms (Kg); whereas, in patients < 15 years old were considered half of the total doses. Similarly, the administration of the antipyretic acetaminophen was costed considering that adults receive 10 tablets (500 mg) and that children take syrup (120 mg/5 ml/) [25, 30]. Follow-up costs considered that *P. vivax* and *P. falciparum* episodes require respectively one and two follow-ups as national guidelines states [25].

#### Patient perspective, direct costs

Direct costs from the patient perspective, comprised other medications not provided free-of-charge by the provider, such as analgesics, the transport costs for the patient and companions, and other costs (e.g. consumed food during the care-seeking process). Cost calculations were based on the volume and unit cost data collected through the socio-economic survey (Table 2).

#### Patient perspective, indirect costs

Indirect costs from the patient perspective included the lost wages due to malaria per patient, substitute labourers and companions. Lost wages were calculated for only individuals ≥ 15 years old only (i.e. economically active sub-population) [32, 33], by multiplying the reported time lost from work in days (cost parameter expressed in person-days) by the amount of money lost in 1 day for not working (person-day unit cost). Person-day unit costs (US$ 9.91) for patients, substitutes and companions were calculated using the monthly minimum wage of 2017 (US$ 257.6) as reference, by dividing it by the number of working days per month [34].

#### Total costs and average cost per episode

The sum of costs from the provider and patient perspectives yielded the average total costs for all malaria episodes during 1 year, and the division between these average total costs by the number of episodes the average cost per episode. Both costs were calculated overall and by species.

### Uncertainty and sensitivity analysis

Multi-way probabilistic sensitivity analysis (PSA) estimated the confidence in the average cost per malaria episode. This considered the uncertainty that surrounded the base case parameters (point estimates) which were used in the calculation of such average cost [35]. Since base case estimates based on few data are more uncertain (less confidence) than estimates based on large datasets, it was accounted by this uncertainty by first defining probability distributions for the relevant cost parameters (Table 2). The rationale for the assumed distribution for each parameter has been described elsewhere [35, 36]. The beta distribution was chosen for the probability of being economically active either among malaria-infected individuals or companions; while the gamma distribution was used for unit costs (i.e. unit costs related to malaria detection, diagnosis and treatment, and costs associated with the transport of patients and their companions) and the time lost from work due to malaria (Table 2). Second, Monte Carlo simulations were conducted in R software to propagate uncertainty by sampling values from all the probability distributions jointly in 10,000 iterations and calculating the corresponding values for the cost per malaria episode. Third, upper and lower 95% uncertainty limits (2.5 and 97.5%) for the average cost per malaria episode were obtained from these simulations and reported using tornado diagrams.

A one-way PSA was also used to assess how sensitive the average cost per species-specific episode was to changes in individual uncertain parameters by sampling from the uncertainty distribution of a single parameter, while fixing the other uncertain parameter to their base case value. The gross domestic product (GDP) of Peru in 2017 [37] was also tested as reference in the sensitivity analysis to estimate person-day unit costs (US$ 21.1) for patients, substitutes and companions, and to adjust the staff-costs associated with the health care services. The latter adjustment consisted in a multiplication by 2.1 (division of person-day unit cost using the GDP reference by that using the monthly minimum wage reference).

## Results

### Household characteristics

A total 680 households and 3877 residents were censused (Table 1). Household with more than three people per habitable room (overcrowding) (57.2%) and housing structures with incomplete walls (60.5%) were frequent in the study area. Most individuals lived in houses with wooden walls and roofs composed of palm leaf. Most households had no sanitation facility and lack of electricity. Main sources of water for domestic use were the river and the rain (Table 3).

**Table 3:** Baseline household characteristics of the study area

Characteristics	N = 680	%
Housing structure (number of external walls)		
0	99	14.6
1–3	312	45.9
4	269	39.6
Main material in walls (n = 581)^a^		
Wood	551	94.8
Brick	2	0.3
Others (adobe, straw, palm)	28	4.8
Main material in floor		
Wood	659	96.9
Cement or other fine finish	12	1.8
Soil	9	1.3
Main material in roof		
Palm leaf, straw	446	65.7
Tin	233	34.3
Missing	1	
Overcrowding		
No	291	42.8
Yes	389	57.2
Sanitation facility		
No facility, field	528	77.9
Pit latrine, ground dug	150	22.1
Missing	2	
Source of water for domestic use		
River, rain	601	88.4
Open well, public tap	79	11.6
Electricity available		
No	473	69.7
Yes	206	30.3
Missing	1	
Radio available		
No	302	44.4
Yes	378	55.6
Ownership of bed nets		
At least one insecticide treated net (ITN) for every two people		
No	453	66.6
Yes	227	33.4
At least one untreated net for every two people		
No	358	52.7
Yes	322	47.4
At least one ITN/untreated net for every two people		
No	109	16.0
Yes	571	84.0
Indoor residual spraying (previous 12 months)		
No	310	46.1
Yes	363	53.9
Missing	7	

^a^Only in household with external walls

### Malaria knowledge, attitudes and practices (KAP)

#### Knowledge

Although most of the 680 responders knew that malaria is transmitted by infected mosquitoes; 22% of responders pointed out that malaria was acquired by drinking stagnant water and nobody knew that malaria could be transmitted through blood transfusion. Fever, headache and chills were identified as the most common symptoms (about 80% of responders) in individuals with malaria disease; myalgia/arthralgia, nausea/vomiting and general discomfort were also recognized as malaria symptoms (> 15% of responders). The majority (> 90%) of responders knew that malaria can be acquired more than once and that medication can cure the disease. Health workers followed by CHW were referred as the main sources of information about malaria (Additional file 1: Table S1).

#### Attitudes

The majority of responders (80.6%) would go to a health facility as first action in case of malaria symptoms, despite the perception (53.5% of responders) that getting malaria was common and normal (Additional file 1: Table S2). The MoH was identified as the main responsible for malaria control (about 60% of respondents), followed by CHW. Almost all responders (> 98%) referred that a finger-prick blood smear was necessary to determine if a person had malaria. Regarding the anti-malarial treatment, about half the responders were aware that interrupting the treatment could lead to a severe illness/death, or to malaria recurrences. The majority of responders perceived that the presence of standing water increased the risk of malaria (88.7%), that the presence of mosquitoes was a nuisance to them (94.3%), and that the use of bed net was not annoying (90.9%).

#### Practices

When they or their relatives had malaria, the majority of responders (79.3%, 525/662) indicated they consulted with health services or with CHW (12.7%, 84/662) (Additional file 1: Table S3). Regarding indoor malaria prevention measures, the most common practices was cleaning the house (51.4%) followed by using bed nets (23.0%), drinking boiled/chlorinated water (22.7%) and covering water containers (19.0%). A majority (58.6%) also responded that cleaning of house surroundings was the main outdoor malaria prevention measure, followed by clearing objects that can accumulate water (23.2%) and vegetation (21.4%).

#### Socio-economic survey

In September 2017, 161 individuals (in 112 households) were enrolled in the socio-economic survey of 251 individuals registered by health services as having had malaria between June–August 2017. Non-enrolled individuals were recent migrants or were not available at household during the survey. Of the total 161 enrolled individuals, 147 (91.3%) individuals presented only one uncomplicated malaria episode, and 14 (8.7%) individuals two malaria episodes. Among the total 175 registered episodes, 124 (70.9%) were by *P. vivax*, 47 (26.9%) by *P. falciparum*, and four (2.3%) co-infections by both parasite species (Additional file 1: Table S4).

Overall, males outnumbered females (ratio male/female = 1.15), and economically active individuals (≥ 15 years) representing about half of total patients reported subsistence farming (81.5%) as the most commonly reported occupation (Table 4). Monthly income in the latter age group ranged from US$ 0 to US$ 121. Moreover, the highest educational attainment of the majority of individuals ≥ 18 years was primary school level (60.3%) with no statistical differences between basins. More than 95% of enrolled individuals had health insurance (i.e. IHIS) and reported having used a bed net the previous night. Canoeing (by dugout canoe “peke-peke”) (47.2%) and walking (47.2%) were the main modes of transportation to reach the point of diagnosis and treatment.

**Table 4:** Socio-demographic characteristics of malaria patient

Characteristics	N = 161	%
Gender		
Female	75	46.6
Male	86	53.4
Age (years)		
1–4	24	14.9
5–14	56	34.8
≥ 15	81	50.3
Education (≥ 18 years) n = 68		
None	8	11.8
Primary school	41	60.3
Secondary school	19	27.9
Main occupation (≥ 15 years) n = 81		
Logger	2	2.5
Farmer	66	81.5
Housewife	3	3.7
Student	6	7.4
Merchant	3	3.7
Distiller	1	1.2
Malaria species		
*P. vivax*	113	70.2
*P. falciparum*	44	27.3
Co-infection (*P. vivax*–*P. falciparum)*	4	2.5
Monthly income amount (≥ 15 years)		
None	10	12.4
US$ 3–121	71	87.6
Type of transportation to the point of diagnosis/treatment		
None (live in the same place)	2	1.2
Foot	76	47.2
Mototaxi/Motorcycle	2	1.2
Dugout canoe	76	47.2
Row-boat	1	0.6
Foot + Mototaxi/Motorcycle	1	0.6
Foot + dugout canoe	2	0.6
Van	1	1.2

Table 5 shows the place to which individuals with malaria sought care for the first time. CHWs (71, 40.6%) were the most consulted, followed by health posts (59, 33.7%), the health centre (43, 24.6%), a hospital (1, 0.6%) and a pharmacy (1, 0.6%). When the first consultation was with CHWs, 40.8% (29/71) of the patients had immediate diagnosis with RDT and treatment, however two patients visited the health centre later (because treatment was not available). The remaining 59.2% (42/71) of the patients had a blood smear taken which was sent to the health centre through an informal fluvial courier for a malaria microscopic diagnosis. With the exception of a single patient who went to the health centre to receive his/her diagnosis and treatment, everyone else came back two-six hours later or the following day for the malaria diagnosis and treatment. Malaria result was communicated by radio to the CHW when radio was available in the community, or sent back through the informal fluvial courier. When the first consultation was at health post, 49.2% (29/59) of the patients had immediate diagnosis with RDT and treatment, and 30 (50.8%) had a blood smear sent to the health centre for malaria microscopic diagnosis. The latter patients came back two-six hours later or the following day for the malaria diagnosis and treatment. Malaria result was communicated by radio to the health posts. The initial seeking for the care at the health centre (43, 24.6%) and at hospital (1, 0.6%) always involved microscopic diagnosis by LM, except in one case diagnosed by RDT at the health centre. An additional individual sought for initial care at the pharmacy, but the malaria diagnosis was done at health centre.

**Table 5:** Characteristics of malaria episodes by diagnosis test

Variable	LM		RDT		Total		Time to diagnosis (days)		Time lost (days)	
							LM	RDT	LM	RDT
	n	%	n	%	n	%	median(IQR)	median(IQR)	median(IQR)	median(IQR)
Consultation (N = 175)										
Health post	30	50.9	29	49.2	59	100				
Health centre	42	97.7	1	2.3	43	100				
Hospital	1	100.0	0	0.0	1	100				
CHW	41	60.3	27	39.7	68	100				
Pharmacy then health centre	1	100.0	0	0.0	1	100				
CHW then health centre	1	33.3	2	66.7	3	100				
Malaria species (N = 175)										
*P. vivax*	76	61.3	48	38.7	124	100	3 (2–7)	3 (2–3)		
*P. falciparum*	40	78.4	11	21.6	51	100	3 (2–7)	2 (2–6)		
Malaria species in EA individuals (N = 85)*										
*P. vivax*	38	69.1	17	30.9	55	100	4.5 (3–7)	3 (2–3)	7.5 (5–15)	7 (4–13)
*P. falciparum*	24	80.0	6	20.0	30	100	3 (2.5–7)	2.5 (2–7)	8.5 (7–16.5)	8.5 (5–14)

CHW, Community health worker; EA, economically active; LM, light microscopy; RDT, rapid diagnostic test; IQR, interquartile range

*p value < 0.05 for differences in time to diagnosis (LM vs RDT)

Only 65 (37.1%) episodes were confirmed within 48 h of the onset of symptoms. Time to diagnosis had a median (mdn) of 3 days and interquartile range (IQR) of 2-5 days, with no differences between malaria episodes by *P. vivax* and *P. falciparum* (Table 5). Considering episodes in only economically active individuals, time to malaria diagnosis was significantly shorter when diagnosis was done by RDT than by microscopy (*p *= 0.01). The analysis by species, showed that these differences were especially for *P. vivax* episodes (*p *= 0.01). However, there were not significant differences in the time lost due to malaria between individuals with episodes diagnosed by RDT and those by microscopy (*p *= 0.50 for *P. vivax*, *p *= 0.62 for *P. falciparum*). Gamma distribution models found a significant association between the reported time to malaria diagnosis and the time lost due to malaria illness in economically active patients, with near twice the time lost due to illness in patients who had the malaria diagnosis after 48 h of the onset of symptoms in comparison who those who had the diagnosis before (Adj. MR 1.8; 95% CI 1.3, 2.6) (Table 6).

**Table 6:** Generalized linear models of time lost due to malaria in economically active patients (≥ 15 years old)

Variable	Univariate				Multivariate	
	N	PD mean (SD)	Mean ratio	[95% CI]	Mean ratio	[95% CI]
Delayed diagnosis						
No (0–48 h)	26	7.2 (7.4)	Ref		Ref	
Yes (> 48 h)	59	12.3 (8.5)	1.7	[1.2;2.4]	1.8	[1.3;2.6]
Diagnosis test						
RDT	23	9.4 (7.4)	Ref		Ref	
Microscopy	62	11.3 (8.7)	1.2	[0.8;1.7]	1.2	[0.8;1.7]
Malaria species						
*P. vivax*	55	10.2 (8.3)	Ref		Ref	
*P. falciparum*	30	11.8 (8.7)	1.2	[0.8;1.7]	1.2	[0.8;1.6]
Gender						
Female	39	11.5 (9.1)	Ref		Ref	
Male	46	10.1 (7.8)	0.9	[0.6;1.2]	0.9	[0.6;1.3]
Education level						
Primary or higher	76	10.3 (8.0)	Ref		Ref	
No education	9	14.3 (11.3)	1.4	[0.8;2.5]	1.6	[1.0;2.8]
Time to the health centre						
< 120 min	46	9.5 (7.1)	Ref		Ref	
≥ 120 min	39	12.3 (9.7)	1.3	[0.9;1.8]	1.2	[0.8;1.7]

PD, person-day; 95% CI, 95% confidence interval; SD, standard deviation

### Costs for uncomplicated malaria episodes

The total costs, for patients and providers combined, associated with 1344 uncomplicated malaria episodes in Mazan in 2017 were US$ 217,006: US$ 160,285 corresponding to 1029 *P. vivax* episodes and US$ 56,721 to 315 *P. falciparum* episodes (Table 7). Therefore, average costs amounted to US$ 161 per average episode or US$ 156 per *P. vivax* episode and US$ 180 per *P. falciparum* episodes. The indirect costs (due to time lost from work) incurred by patients contribute to 78% of the total costs (Table 7).

**Table 7:** Total cost from both patient and provider perspectives for uncomplicated malaria episodes in Mazan district 2017 (US$)

Perspective	*P. vivax* (n = 1,029)				*P. falciparum* (n = 315)				Total (N = 1344)					
	Monthly minimum wage		GDP per capita		Monthly minimum wage		GDP per capita		Monthly minimum wage			GDP per capita		
	Sub-total costs	Cost per uncomplicated episode	Sub-total costs	Cost per uncomplicated episode	Sub-total costs	Cost per uncomplicated episode	Sub-total costs	Cost per uncomplicated episode	Total cost	Cost per uncomplicated episode	%	Total cost	Cost per uncomplicated episode	%
Provider														
Direct cost	29,450.57	28.62	49,861.25	48.46	12,014.98	38.14	19,126.51	60.72	41,465.55	30.85	19.1	68,987.8	51.3	15.8
Patient														
Direct cost	4723.11	4.59	4723.11	4.59	1124.55	3.57	1124.55	3.57	5847.66	4.35	2.7	5847.7	4.4	1.3
Indirect cost	126,111.69	122.56	268,003.24	260.45	43,581.01	138.35	92,615.14	294.02	169,692.70	126.26	78.2	360,618.4	268.3	82.8
Direct and indirect costs	130,834.80	127.15	272,726.35	265.04	44,705.56	141.92	93,739.69	297.59	175,540.36	130.61	80.9	366,466.0	272.7	84.2
Total cost	160,285.36	155.77	322,587.60	313.50	56,720.54	180.07	112,866.20	358.31	217,005.91	161.46	100	435,453.8	324.0	100

Monthly minimum wage 2017: US$257.6; GDP (Gross domestic product) per capita 2017:US$ 6,571.9

From the provider perspective, estimated costs in Mazan were US$ 41,466 (US$ 30.85 per episode), including US$ 29,451 associated with 1029 *P. vivax* episodes (US$ 28.62 per episode) and US$ 12,015 with 315 *P. falciparum* episodes (US$ 38.14 per episode). As Additional file 1: Tables S5 and S6 show, costs associated with initial consultations and malaria screenings (by LM or RDT) accounted for most of provider costs for both *P. vivax* (76.40%) and *P. falciparum* (57.35%). Moreover, costs of anti-malarial treatment and follow-up control after treatment for one *P. falciparum* episode (US$ 13.13 and US$ 6.41) were higher than those for one *P. vivax* episode (US$ 1.98 and US$ 3.20).

From the patient perspective, estimated costs for uncomplicated malaria episodes in Mazan were US$ 175,540 (US$ 131 per episode), including US$ 5848 (US$ 4.35 per episode) as direct costs and US$ 169,693 (US$ 126 per episode) as indirect costs. The analysis by species showed that the average costs per *P. falciparum* episode (US$ 142) were higher than those per *P. vivax* episode (US$ 127) (Table 7). The longer time lost by patients due to *P. falciparum* episodes (22.20 days) in comparison with *P. vivax* episodes (17.0 days) determined the higher indirect costs for *P. falciparum* episodes (US$ 138 per episode) in comparison with those for *P. vivax* episodes (US$ 123 per episode) (Additional file 1: Tables S7 and S8).

### Uncertainty and sensitivity analysis

**Fig. 2:**
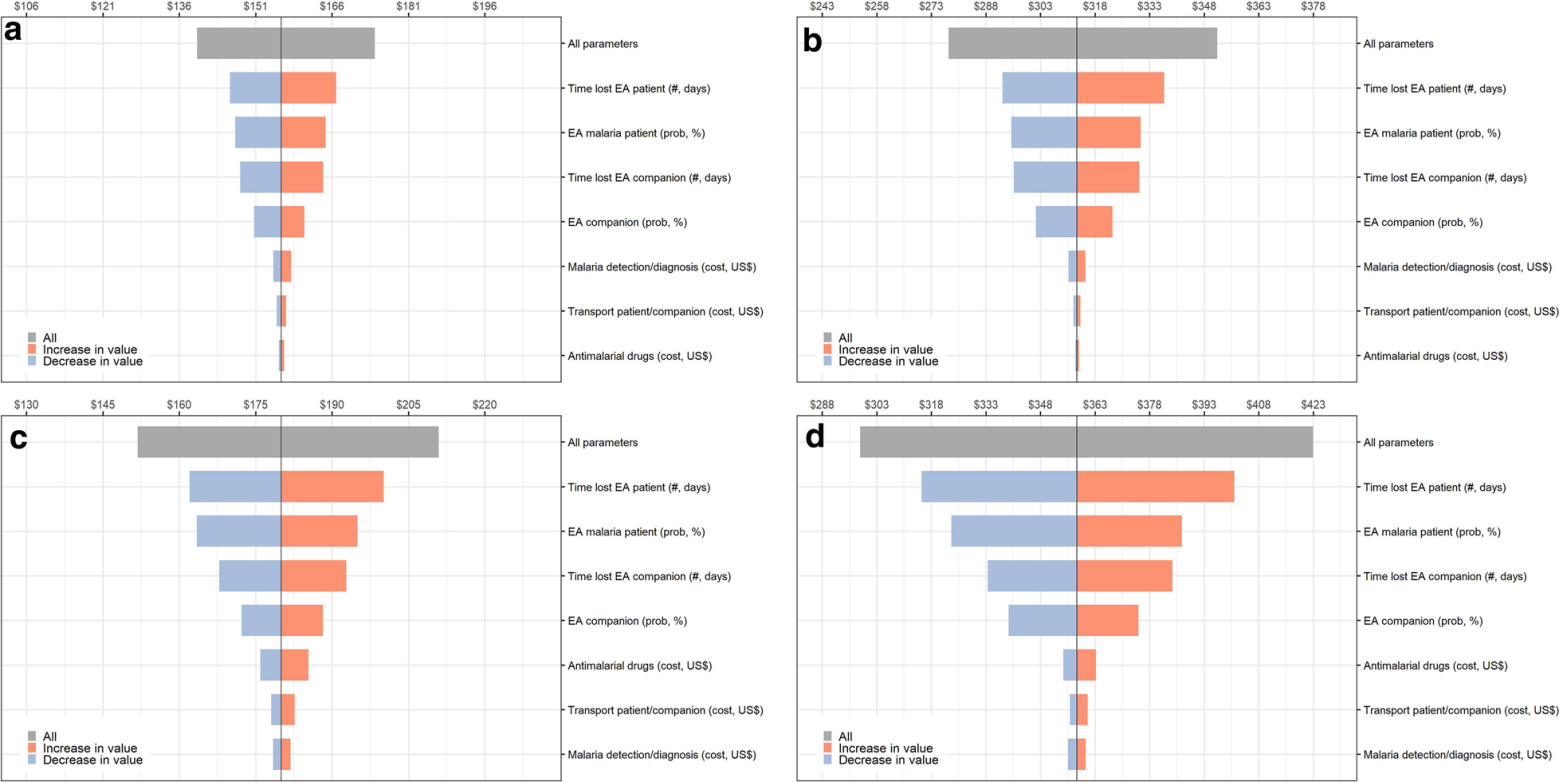
Tornado diagram showing results of probabilistic sensitivity analysis (PSA) exploring the effect of key parameters on average costs per uncomplicated malaria episode in Mazan, 2017:** a***P. vivax* episode, monthly minimum wage as reference to estimate person-day costs,** b***P. vivax* episode, gross domestic product as reference to estimate person-day costs gross domestic product, **c***P. falciparum* episode, monthly minimum wage as reference to estimate person-day costs, **d***P. falciparum* episode, gross domestic product as reference to estimate person-day costs. One-way PSA (example: the lower the time lost in economically active (EA) patient, blue color, the lower the average costs per uncomplicated malaria episode); Multi-way PSA (gray color)

Multi-way PSA, accounting for the uncertainty of all relevant cost parameters at the same time and considering the monthly minimum wage as reference, found confidence intervals (95% CI) for the average cost per episode between US$ 139 and US$ 174 for *P. vivax*, and between US$ 152 and US$ 211 for *P. falciparum* (gray bars in Fig. 2a, c).

According to the one-way PSA, the uncertainty about the four parameters related to the cost of loss of productivity due to malaria, induced most of the uncertainty in the average cost per malaria episode (second, third, fourth and fifth coloured bars in Fig. 2a, c). Of these four, the uncertainty about the time lost due to malaria in an economically active patient had most impact on the average cost per episode, with estimates between US$ 146 and US$ 167 per *P. vivax* episode, and between US$ 162 and US$ 200 per *P. falciparum* episode. The uncertainty about the proportion of patients ≥ 15 years (economically active individuals) was less influential, with estimates of average cost per episode between US$ 147 and US$ 165 per *P. vivax* episode, and between US$ 164 and US$ 195 *per P. falciparum* episode. Similarly, the uncertainty about having economically active companions resulted in average costs between US$ 150 and US$ 160 per *P. vivax* episode, and between US$ 172 and US$ 188 per *P. falciparum* episode. But, again the uncertainty in the time lost in economically active companions during the healthcare-seeking, malaria diagnosis and treatment of a malaria patient caused more uncertainty in the average cost estimates: between US$ 148 and US$ 164 per *P. vivax* episode and between US$ 168 and US$ 193 per *P. falciparum* episode. Uncertainties about the cost of anti-malarial drugs, the cost of malaria detection and diagnosis, and the cost of transport resulted in the smallest uncertainty intervals of the average costs per episode.

When GDP was used as reference to estimate person-day unit costs, the base case average costs increased to US$ 314 *per P. vivax* episode, and to US$ 358 *per P. falciparum* episode (Table 7). Multi-way PSA found averages cost ranges between US$ 278 and US$ 352 *per P. vivax* episode, and between US$ 299 and US$ 423 *per P. falciparum* episode (gray bars in Fig. 2b, d). The most influential parameters for the average cost per malaria episode were also those that determined the cost of loss of productivity due to malaria according to the one-way PSA.

## Discussion

This research provides total costs and average costs per episode associated with the detection and management of uncomplicated malaria in Mazan, an exemplar district in the Peruvian Amazon where poverty, limited accessibility from communities to health facilities, and healthcare-seeking behaviour challenge malaria control efforts. Estimated total costs for the 1344 uncomplicated malaria episodes registered in Mazan in 2017 were US$ 217,006 with most of these costs (78%) corresponding to indirect costs incurred by patients. Average costs per episode were higher for *P. falciparum* (US$ 180) than for *P. vivax* (US$ 156), mainly due to differences in costs of the anti-malarial treatment and in time losses due to illness. Indeed, the length of time lost due to illness in a patient, found to be associated with delayed confirmatory malaria diagnosis, was the parameter that caused most uncertainty in the average costs per malaria episode for both species.

As in Colombia [38], Brazil [39] and other endemic areas outside the Americas [19, 40, 41], uncomplicated malaria episodes in Mazan imposes significant costs on households, pushing poor families further into poverty. In several Amazonian communities with high risk of malaria transmission, the situation can become catastrophic due to the occurrence of repeated episodes in the same individuals and/or the same households every year [11, 38, 39, 42, 43]. In contrast to the low costs of transport and medications paid out of pocket by families, indirect costs of malaria episodes were the key determinant of the disease’s overall costs. This is because adults have to interrupt their normal activities to care for ill family members, or the disease directly afflicts the economically active population [11, 40, 44].

The indirect costs of a *P. vivax* episode is markedly higher in Mazan in 2017 (US$ 260.45 with GDP; US$ 122.56 with minimum wage) than in Afghanistan, Ethiopia, Indonesia and Vietnam in 2016 [45] and Papua New Guinea in 2013 [45]. This is partially due to Peru having a higher GDP per capita, which was used to estimate the lost wages of patients and their carers/companions. Specifically, in Papua, Indonesia, malaria associated costs may have been underestimated since they were calculated based on data collected in localities with high access to health facilities by road [40]. In the study area, the limited access to health facilities would also have increased the time lost due to illness with corresponding uncertainty for economically active patients and carers/companions during the period in which healthcare is sought for diagnosis, treatment and follow-up. As a result, the costs of case management of one *P. vivax* episode in Mazan (US$ 313.50 based on GDP per capita, US$ 155.77 based on minimum wages) were higher than the estimates in Papua, Indonesia (US$ 44.50 based on mean wage) [40].

The association between longer time losses in a malaria patient and delayed malaria diagnosis could reflect a more compromised clinical case (without the need for hospitalization) that requires more recovery time. Indeed, several studies have shown an increase in malaria severity with a delay in malaria case management [20, 46, 47], thus the WHO’s recommendation of insuring access to early diagnosis, and prompt, effective treatment within 24-48 h of the onset of malaria symptoms [30]. The reduced group of participants (37.1%) with malaria diagnosis within 48 h of onset of symptoms suggests that the delay in the malaria diagnosis remains a pitfall of the malaria case management in the rural and widely dispersed population of the Peruvian Amazon, as previously found by a longitudinal study (2001–2003) in riverine and road-associated rural populations of peri-Iquitos districts [48, 49].

It was not surprising to find that the availability of RDTs in the first place to which enrolled participants sought for healthcare (i.e. CHW or health posts) reduced the time from the onset of symptoms to malaria diagnosis. The impact of incorporating the use of RDTs to get timely diagnosis and appropriate treatment has already been demonstrated in rural Amazonian communities [48, 49]. Where RDTs were not available in the study communities, unreliable or irregular fluvial transport and limited access to the health centre in MT may have delayed any patient reference and/or sending of blood smear samples for malaria confirmation [50]. However the findings show that even with an easy access to malaria diagnosis, an early malaria case management of participants was not guaranteed [48]. The time to diagnosis of three or more days in several participants who went directly to health facility or CHW with RDTs available, suggest a delay in seeking care. The community’s knowledge, misperceptions (e.g. perceiving malaria as a common, uneventful, and a mild and self-limiting disease), the lack of household involvement in community control of malaria, and initial self-treatment (e.g. use of antipyretics and refreshing herbs at home) reported in the KAP survey may be associated with such delay to seek care [51–53].

Several studies have highlighted the need for economic research on the household costs of illness, household responses, and their implications for poverty [44]. The lack of knowledge about the economic burden of illness for households and/or the poor understanding about the effects on the quality of life and well-being in the population may lead health stakeholders to plan and deliver health services with the main goals of increasing coverage and reducing disease burden. This decision-making process may be more obvious in the case of services that are provided free-of-charge like the detection, diagnosis and treatment of malaria provided by the Peruvian MoH in the entire country [54, 55]. Increased access to prevention measures (e.g. delivery of long lasting insecticide treated bed nets) and malaria behaviour change communication (BCC) strategies would remain as the best options to avoid indirect and direct household costs due to malaria.

Similar to other studies conducted in Peru [11, 49], the provider in Mazan had to use more resources for delivering diagnosis and treatment services to an individual with a *P. falciparum* episode than one with a *P. vivax* episode. This was also observed in Papua, Indonesia, where the cost differences between both species were associated with additional consultations, transportation to the health facility and lost wages per episode [40]. The difference in costs of first-line treatment regimens between species (US$ 8.76 for *P. falciparum* and US$ 1.32 for *P. vivax*) explains well the higher provider costs of a *P. falciparum* episode. Delays in adjusting the daily dosage of one of the drugs (MQ) that make up the anti-malarial combined regimen for *P. falciparum* (MQ + AS + PQ) according to national policies have made difficult and expensive the procurement of separate presentations of these drugs because low volumes are less interesting for pharmaceutical companies, ceteris paribus. These delays have also prevented the purchase of cheaper drug presentations (AS-MQ fixed dose combinations instead of separate drug blisters) [56], which are used in other South-American countries such as Bolivia, Brazil and Venezuela [57, 58]. The uncertainty in the unit cost of the anti-malarial treatment for *P. falciparum* likely depends on these procurement issues.

This study has a number of limitations. First, budget constraints did not allow to include all the 70 communities of Mazan and to visit households more than three times to maximize participation in the socio-economic survey; however, the chosen communities (accounting for 80% of malaria incidence) and the cost parameters obtained from the survey likely reflect the case-management´s situation in the district. Second, verbal report of time losses due to illness and costs may affect the accuracy of the cost estimates from the patient perspective. This possibility was reduced with a short recall. Third, productivity changes may occur because of absence from work (absenteeism) or because of reduced productivity while at work (presenteeism). This information may be derived from existing data sources, such as registrations from occupational health service companies. Nonetheless, such registrations are typically available only for salaried workers and not for self-employed workers, such as subsistence farmers [59]. Fourth, from a patient perspective, indirect costs for children were considered zero as they should not be economically active. However, children represent a risk group and as 41% of the population were children, the methods for valuing the productivity costs could lead to biased estimates; this could be due to educational losses. Fifth, malaria infection can cause macroeconomic costs, which cannot be assessed in this study at the household level. For example, malaria can affect foreign direct investments, international trade and tourism. Sixth, mental stress and social costs of families with sick members were not included, which are in general very difficult to evaluate over a short period of time (and are typically not monetised in health economic evaluations). Finally, as this study was focused on uncomplicated malaria, costs may have been underestimated due to the exclusion of the cost of primaquine-induced haemolysis and the cost of adverse effects. Primaquine-induced haemolysis was not included in any of the estimates due to uncertainty related to its frequency and severity. In countries that are prescribing primaquine without testing for glucose-6-phosphate dehydrogenase (G6PD) deficiency, as in Peru, it is possible that haemolysis already occurs, however in previous studies, Peru has reported low (< 2%) G6PD deficiency [60].

## Conclusions

Access to prompt diagnosis and treatment is one of the principal strategies for malaria control and elimination. This includes two components, one dependent upon the patient to seek care when sick and the other being the availability of accessible diagnosis and treatment facilities. Although malaria case management is provided free-of-charge by the MoH in Peru, this study confirmed that malaria poses a significant economic burden on rural households and individuals in terms of indirect costs due to the loss of working days. These costs are not well perceived by society and the substantial financial impact of the disease may be undervalued in public policy planning. Malaria control policies need to be integrated into development and poverty reduction programmes as socioeconomic development may be an effective and sustainable intervention against malaria in the long term.

## Supplementary information

**Additional file 1: Table S1.** Knowledge of household heads on malaria. **Table S2.** Attitudes of household heads on malaria. **Table S3.** Malaria prevention practices among household heads. **Table S4.** Malaria episodes and household by basin. **Table S5.** Direct cost from the provider perspective for uncomplicated malaria by *P. vivax* in Mazan district 2017 (US$). **Table S6.** Direct cost from the provider perspective for uncomplicated malaria by *P. falciparum* in Mazan district 2017 (US$). **Table S7.** Direct and indirect costs from the patient perspective for uncomplicated malaria by *P. vivax* in Mazan district 2017 (US$). **Table S8.** Direct and indirect costs from the patient perspective for uncomplicated malaria by *P. falciparum* in Mazan district 2017 (US$).

## Data Availability

All data generated during this study are included in this published article and its Additional file 1.

## Abbreviations

ACD: Active case detection
API: Annual parasite index
AS: Artesunate
BCC: Behaviour change communication
BH: Bello Horizonte
CI: Confidence interval
CJ: Catorce de Julio
CHW: Community health workers
CQ: Chloroquine
EsSalud: Social health insurance system
FFAA: Armed Forces
GDP: Gross Domestic Product
G6PD: Glucose-6-phosphate dehydrogenase
GNI: Gross national income
HRP-II: Histidine-rich protein II
HU: Huaman-Urco
IHIS: Integrated Health Insurance Scheme
KAP: Knowledge, attitudes and practices
LI: Libertad
LM: Light microscopy
MA: Mangua
MQ: Mefloquine
MT: Mazan town
MoH: Ministry of Health
MDGs: Millennium Development Goals
NMCP: National Malaria Control Program
ODK: Open Data Kit
PCD: Passive case detection
pLDH: *Plasmodium* lactate dehydrogenase
PQ: Primaquine
PAB: Puerto Abeja
PA: Puerto Alegre
PE: Primero de Enero
PEN: Peruvian Nuevos Soles
PO: Puerto Obrero
PU: Puinahua
PNP: National Police
RDT: Rapid diagnostic test
SA: Salvador
SZ: San Antonio de Zambrano
SC: Santa Cruz
SF: San Francisco de Buen Paso
SU: Sucusari
SDGs: Sustainable Development Goals
TA: Tamanco
US$: United States Dollars
UM: Urco Miraño
VB: Visto Bueno
WHO: World Health Organization
