# Supplementary material for: Economic costs analysis of uncomplicated malaria case management in the Peruvian Amazon
Source: Malar J. 2020 Apr 21;19:161. doi: 10.1186/s12936-020-03233-5 (PMC7175533; doi:10.1186/s12936-020-03233-5)
Supplement: Supplementary file 1 — Additional file 1: Table S1. Knowledge of household heads on malaria. Table S2. Attitudes of household heads on malaria. Table S3. Malaria prevention practices among household heads. Table S4. Malaria episodes and household by basin. Table S5. Direct cost from the provider perspective for uncomplicated malaria by P. vivax in Mazan district 2017 (US$). Table S6. Direct cost from the provider perspective for uncomplicated malaria by P. falciparum in Mazan district 2017 (US$). Table S7. Direct and indirect costs from the patient perspective for uncomplicated malaria by P. vivax in Mazan district 2017 (US$). Table S8. Direct and indirect costs from the patient perspective for uncomplicated malaria by P. falciparum in Mazan district 2017 (US$). [file 12936_2020_3233_MOESM1_ESM.docx]

SUPPLEMENTARY INFORMATION

| **Table S1. Knowledge of household heads on malaria** | |  |  |
| --- | --- | --- | --- |
|  |  |  |  |
| Variable (Responded affirmatively) | | N=680 | % |
|  |  |  |  |
| ***Knowledge*** | |  |  |
| Mode of transmissionǂ | |  |  |
|  | Mosquito bite | 501 | 73.9 |
|  | Contact with malaria patient | 15 | 2.2 |
|  | Drinking stagnant water | 149 | 22.0 |
|  | Contact with animals | 1 | 0.2 |
|  | Blood transfusion, syringes contaminated with blood | 0 | 100 |
|  |  |  |  |
| Symptomsǂ | |  |  |
|  | Fever | 588 | 86.7 |
|  | Chills | 518 | 76.4 |
|  | Headache | 572 | 84.4 |
|  | Profuse sweating | 20 | 3.0 |
|  | Myalgia/arthralgia | 381 | 56.2 |
|  | General discomfort | 121 | 17.9 |
|  | Nausea and vomiting | 190 | 28.0 |
|  | Diarrhea | 24 | 3.5 |
|  | Abdominal pain | 36 | 5.3 |
|  |  |  |  |
| Malaria can be cured with medicaments | |  |  |
|  | No | 38 | 5.9 |
|  | Yes | 611 | 94.1 |
|  | Missing | 31 |  |
|  |  |  |  |
| A patient can have malaria more than once | |  |  |
|  | No | 17 | 2.5 |
|  | Yes | 655 | 97.5 |
|  | Missing | 8 |  |
|  |  |  |  |
| Source of informationǂ | |  |  |
|  | Friends, neighbours | 50 | 7.4 |
|  | School | 8 | 1.2 |
|  | Health workers | 430 | 63.4 |
|  | Radio or television | 61 | 9.0 |
|  | Community health worker | 153 | 22.6 |
|  | My experience/ I suffered from malaria | 78 | 11.5 |
|  | Non-governmental organization | 12 | 1.8 |
|  | Family | 24 | 3.5 |
|  | Poster | 8 | 1.2 |
| ǂMore than one response was possible for these questions | | | |

| **Table S2. Attitudes of household heads on malaria** | | | |
| --- | --- | --- | --- |
|  |  |  |  |
| Variable (Responded affirmatively) | | N=680 | % |
|  |  |  |  |
| ***Attitude*** | |  |  |
| First action when you have symptoms of malariaǂ | |  |  |
|  | Go to a health centre | 547 | 80.6 |
|  | Go to a community health worker | 97 | 14.3 |
|  | Go to pharmacy | 6 | 0.9 |
|  | Go to shop | 5 | 0.7 |
|  | Self‑treat at home drinking herbs | 37 | 5.5 |
|  | Go to traditional healers | 0 | 100 |
|  |  |  |  |
| Getting malaria is common and normal | |  |  |
|  | No | 309 | 46.5 |
|  | Yes | 355 | 53.5 |
|  | Missing | 16 |  |
|  |  |  |  |
| Responsible for malaria controlǂ | |  |  |
|  | Health centre/Ministry of health | 419 | 61.6 |
|  | Community health worker | 264 | 38.8 |
|  | Myself | 51 | 7.5 |
|  | Community | 6 | 0.9 |
|  |  |  |  |
| Blood smear is neccesary to diagnosis | |  |  |
|  | No | 9 | 1.3 |
|  | Yes | 664 | 98.7 |
|  | Missing | 7 |  |
|  |  |  |  |
| Without complete antimalarial treatmentǂ | |  |  |
|  | Malaria can lead to severe illness and death | 344 | 50.7 |
|  | Malaria can return | 336 | 49.6 |
|  | Transmit to your relatives | 10 | 1.5 |
|  | Malaria cannot be cured | 96 | 14.2 |
|  | Symptoms remain | 24 | 3.5 |
|  | I become weak | 11 | 1.6 |
|  | Nothing happens | 3 | 0.4 |
|  |  |  |  |
| Standing water increase risk of malaria | |  |  |
|  | No | 74 | 11.3 |
|  | Yes | 583 | 88.7 |
|  | Missing | 23 |  |
|  |  |  |  |
| The use of bed net is annoying | |  |  |
|  | No | 617 | 90.9 |
|  | Yes | 62 | 9.1 |
|  | Missing | 1 |  |
|  |  |  |  |
| The presence of mosquitoes bother me | |  |  |
|  | No | 39 | 5.7 |
|  | Yes | 641 | 94.3 |
| ǂMore than one response was possible for these questions | | | |

| **Table S3. Malaria prevention practices among household heads** | | | | |
| --- | --- | --- | --- | --- |
|  |  |  |  |  |
| Variable (Responded affirmatively) | | N=680 | % |  |
|  |  |  |  |  |
| ***Practices*** | |  |  |  |
| First action when you or your relatives had malaria | |  |  |  |
|  | Go to a health centre | 525 | 79.3 |  |
|  | Go to a community health worker | 84 | 12.7 |  |
|  | Self‑treat at home drinking herbs | 36 | 5.4 |  |
|  | Go to drugstore | 3 | 0.5 |  |
|  | Go to shop | 4 | 0.6 |  |
|  | Other | 10 | 1.5 |  |
|  | Missing | 18 |  |  |
|  |  |  |  |  |
| Indoor malaria prevention measuresǂ | |  |  |  |
|  | Use bed net | 156 | 23.0 |  |
|  | Use net for windows and doors | 2 | 0.3 |  |
|  | Fumigation | 26 | 3.8 |  |
|  | Use mosquito repellent | 8 | 1.2 |  |
|  | Cleans the house | 349 | 51.4 |  |
|  | Do nothing | 28 | 4.1 |  |
|  | Drink boiled/chlorinated water | 154 | 22.7 |  |
|  | Cover water containers | 129 | 19.0 |  |
|  | Hand washing | 15 | 2.2 |  |
|  | Wear long-sleeved clothing and long trousers | 17 | 2.5 |  |
|  |  |  |  |  |
| Outdoor malaria prevention measuresǂ | |  |  |  |
|  | Clean surroundings | 397 | 58.6 |  |
|  | Wear long-sleeved clothing and long trousers | 51 | 7.5 |  |
|  | Clear the vegetation | 145 | 21.4 |  |
|  | Drain stagnant water | 74 | 10.9 |  |
|  | Fumigation | 4 | 0.6 |  |
|  | Use mosquito repellent | 7 | 1.0 |  |
|  | Throw any objects that can accumulate water | 157 | 23.2 |  |
|  | Take a shower early | 30 | 4.4 |  |
|  | Do nothing | 35 | 5.2 |  |
| ǂMore than one response was possible for these questions | | | |  |

| **Table S4. Malaria episodes and household by basin** | | | | | | | | | | | | | | | | |
| --- | --- | --- | --- | --- | --- | --- | --- | --- | --- | --- | --- | --- | --- | --- | --- | --- |
|  |  |  |  |  |  |  |  |  |  |  |  |  |  |  |  |  |
|  | **Communities by basin** | **Malaria patients** | |  | **Malaria episodes** | | | | | | |  |  | **Household** | |  |
|  |  |  |  |  | ***P. vivax*** | | ***P.falciparum*** | | **Co-infection*** | | **Total** | |  |  |  |  |
|  |  | **N** | **%** |  | **n** | **%** | **n** | **%** | **n** | **%** | **N** | **%** |  | **N** | **%** |  |
| Mazan | |  |  |  |  |  |  |  |  |  |  |  |  |  |  |  |
|  | Primero de Enero | 14 | 8.7 |  | 15 | 8.6 | 2 | 1.1 | 0 | 0.0 | 17 | 9.7 |  | 8 | 7.1 |  |
|  | Visto Bueno | 15 | 9.3 |  | 9 | 5.1 | 6 | 3.4 | 0 | 0.0 | 15 | 8.6 |  | 9 | 8.0 |  |
|  | 14 de Julio | 6 | 3.7 |  | 4 | 2.3 | 2 | 1.1 | 0 | 0.0 | 6 | 3.4 |  | 4 | 3.6 |  |
|  | Santa Cruz | 37 | 23.0 |  | 33 | 18.9 | 5 | 2.9 | 1 | 0.6 | 39 | 22.3 |  | 25 | 22.3 |  |
|  | Libertad | 30 | 18.6 |  | 20 | 11.4 | 13 | 7.4 | 1 | 0.6 | 34 | 19.4 |  | 22 | 19.6 |  |
|  | Puerto Alegre | 20 | 12.4 |  | 15 | 8.6 | 8 | 4.6 | 1 | 0.6 | 24 | 13.7 |  | 14 | 12.5 |  |
| **Subtotal** | | **122** | **75.8** |  | **96** | **54.9** | **36** | **20.6** | **3** | **1.7** | **135** | **77.1** |  | **82** | **73.2** |  |
| Napo |  |  |  |  |  |  |  |  |  |  |  |  |  |  |  |  |
|  | Huaman Urco | 15 | 9.3 |  | 8 | 4.6 | 7 | 4.0 | 1 | 0.6 | 16 | 9.1 |  | 11 | 9.8 |  |
|  | San Antonio de Zambrano | 4 | 2.5 |  | 3 | 1.7 | 1 | 0.6 | 0 | 0.0 | 4 | 2.3 |  | 2 | 1.8 |  |
|  | Urco Miraño | 2 | 1.2 |  | 1 | 0.6 | 1 | 0.6 | 0 | 0.0 | 2 | 1.1 |  | 2 | 1.8 |  |
|  | Bello Horizonte | 3 | 1.9 |  | 3 | 1.7 | 0 | 0.0 | 0 | 0.0 | 3 | 1.7 |  | 2 | 1.8 |  |
|  | Puerto Abeja | 1 | 0.6 |  | 1 | 0.6 | 0 | 0.0 | 0 | 0.0 | 1 | 0.6 |  | 1 | 0.9 |  |
|  | Tamanco | 1 | 0.6 |  | 0 | 0.0 | 1 | 0.6 | 0 | 0.0 | 1 | 0.6 |  | 1 | 0.9 |  |
|  | Puerto Obrero | 4 | 2.5 |  | 4 | 2.3 | 0 | 0.0 | 0 | 0.0 | 4 | 2.3 |  | 4 | 3.6 |  |
|  | Salvador | 5 | 3.1 |  | 4 | 2.3 | 1 | 0.6 | 0 | 0.0 | 5 | 2.9 |  | 4 | 3.6 |  |
|  | Sucusari | 4 | 2.5 |  | 4 | 2.3 | 0 | 0.0 | 0 | 0.0 | 4 | 2.3 |  | 3 | 2.7 |  |
| **Subtotal** | | **39** | **24.2** |  | **28** | **16.0** | **11** | **6.3** | **1** | **0.6** | **40** | **22.9** |  | **30** | **26.8** |  |
| **Total** | | **161** | **100.0** |  | **124** | **70.9** | **47** | **26.9** | **4** | **2.3** | **175** | **100.0** |  | **112** | **100.0** |  |
| **P.falciparum-P.vivax* | |  |  |  |  |  |  |  |  |  |  |  |  |  |  |  |

| **Table S5. Direct cost from the provider perspective for uncomplicated malaria by *P.vivax* in Mazan district 2017 (US$)** | | | | | |
| --- | --- | --- | --- | --- | --- |
| **Cost category (Units)** | | | **Quantity** | **Unit costs (US$)** | **Total costs (US$)** |
| ***P.vivax* (n=1029)** | | |  |  |  |
| **Initial consultations** | | |  |  |  |
|  | Screnned individuals | |  |  |  |
|  |  | By HF | 5,538 | 2.58 | 14,288.04 |
|  |  | By CHW | 1,100 | 0.62 | 684.95 |
|  | Sub-total | | 6,638 |  | 14,972.99 |
| **Malaria diagnosis** | | |  |  |  |
|  | Screenned individuals by HF | |  |  |  |
|  |  | With LM | 4,400 | 0.71 | 3,124.00 |
|  |  | With RDT | 1,138 | 1.97 | 2,238.45 |
|  |  |  |  |  |  |
|  | Screenned individuals by CHW | |  |  |  |
|  |  | With LM | 0 | 0.71 | 0.00 |
|  |  | With RDT | 1,100 | 1.97 | 2,163.70 |
|  | Sub-total | | 6,638 |  | 7,526.15 |
| **Treatment consultations** | | |  |  |  |
|  |  | By HF | 858 | 2.58 | 2,213.64 |
|  |  | By CHW | 171 | 0.62 | 106.48 |
|  | Sub-total | | 1,029 |  | 2,320.12 |
| **Anti-malarial treatment** | | |  |  |  |
|  | Complete treatment (CQ+PQ) | |  |  |  |
|  |  | Adults | 580 | 1.32 | 766.76 |
|  |  | Children | 449 | 0.66 | 296.79 |
|  | Sub-total | | 1,029 |  | 1,063.55 |
| **Antipyretic drug** | | |  |  |  |
|  | Acetaminophen (doses for 2 days) | |  |  |  |
|  |  | Adults | 580 | 0.09 | 52.20 |
|  |  | Children | 449 | 0.49 | 220.01 |
|  | Sub-total | | 1,029 |  | 272.21 |
| **Follow-up control** | | |  |  |  |
|  | Control | | 1,029 | 3.20 | 3,295.56 |
|  | Sub-total | | 1,029 | 3.20 | 3,295.56 |
| **Total (US$)** | |  |  |  | **29,450.57** |
| Abbreviations: HF (Health facility), CHW (Community Health Worker), LM (Light MIcroscopy), RDT (Rapid Diagnostic Test), CQ (Chloroquine), PQ (Primaquine). | | | | | |

| **Table S6. Direct cost from the provider perspective for uncomplicated malaria by *P.falciparum* in Mazan district 2017 (US$)** | | | | | |
| --- | --- | --- | --- | --- | --- |
| **Cost category (Units)** | | | **Quantity** | **Unit costs (US$)** | **Total costs (US$)** |
| ***P. falciparum* (n=315)** | | |  |  |  |
| **Initial consultations** | | |  |  |  |
|  | Screnned individuals | |  |  |  |
|  |  | By HF | 1,696 | 2.58 | 4,375.68 |
|  |  | By CHW | 337 | 0.62 | 209.84 |
|  | Sub-total | | 2,033 |  | 4,585.52 |
| **Malaria diagnosis** | | |  |  |  |
|  | Screenned individuals by HF | |  |  |  |
|  |  | With LM | 1,348 | 0.71 | 957.08 |
|  |  | With RDT | 348 | 1.97 | 684.52 |
|  |  |  |  |  |  |
|  | Screenned individuals by CHW | |  |  |  |
|  |  | With LM | 0 | 0.71 | 0.00 |
|  |  | With RDT | 337 | 1.97 | 662.88 |
|  | Sub-total | | 2,033 |  | 2,304.48 |
| **Treatment consultations** | | |  |  |  |
|  |  | By HF | 263 | 2.58 | 678.54 |
|  |  | By CHW | 52 | 0.62 | 32.38 |
|  | Sub-total | | 315 |  | 710.92 |
| **Anti-malarial treatment** | | |  |  |  |
|  | Complete treatment (AS+MQ+PQ) | |  |  |  |
|  |  | Adults | 217 | 8.76 | 1,899.84 |
|  |  | Children | 98 | 4.38 | 429.00 |
|  | Sub-total | | 315 |  | 2,328.83 |
| **Antipyretic drug** | | |  |  |  |
|  | Acetaminophen (doses for 2 days) | |  |  |  |
|  |  | Adults | 217 | 0.09 | 19.53 |
|  |  | Children | 98 | 0.49 | 48.02 |
|  | Sub-total | | 315 |  | 67.55 |
| **Follow-up control** | | |  |  |  |
|  | Control | | 315 | 6.41 | 2,017.69 |
|  | Sub-total | | 315 | 6.41 | 2,017.69 |
| **Total (US$)** | |  |  |  | **12,014.98** |
| Abbreviations: HF (Health facility), CHW (Community Health Worker), LM (Light MIcroscopy), RDT (Rapid Diagnostic Test), AS (Artesunate), MQ (Mefloquine), PQ (Primaquine). | | | | | |

| **Table S7. Direct and indirect costs from the patient perspective for uncomplicated malaria by *P.vivax* in Mazan district 2017 (US$)** | | | | | | | | |  |
| --- | --- | --- | --- | --- | --- | --- | --- | --- | --- |
| **Cost category** | | | Unit | Number of episodes | Average person-days lost | Quantity | Unit costs | Sub-total costs (US$) | |
|  |  |  |  |  |  |  |  |  |  |
|  |  |  |  |  |  |  |  |  |  |
| ***P. vivax*** *(n=1029)* | | |  |  |  |  |  |  | |
|  | **Direct costs** | |  |  |  |  |  |  | |
|  |  | Medicaments | Per episode | 1029 |  | 1029 | 0.00 | 0.00 | |
|  |  | Transport cost for patient | Round trip | 1029 |  | 1029 | 2.36 | 2,428.44 | |
|  |  | Transport cost for companions | Round trip | 1029 |  | 1029 | 1.94 | 1,996.26 | |
|  |  | Other cost | Per episode | 1029 |  | 1029 | 0.29 | 298.41 | |
|  |  | **Total direct costs** |  |  |  |  |  | **4,723.11** | |
|  |  |  |  |  |  |  |  |  | |
|  | **Indirect costs** | |  |  |  |  |  |  | |
|  |  | Lost wages for patient |  |  |  |  |  |  | |
|  |  | 1 - 4 years old | Person-day | 181 | 2.7 | 492 | 0.00 | 0.00 | |
|  |  | 5 – 14 years old | Person-day | 268 | 4.1 | 1096 | 0.00 | 0.00 | |
|  |  | >=15 years old | Person-day | 580 | 10.2 | 5922 | 9.91 | 58,685.04 | |
|  |  |  |  |  |  |  |  |  | |
|  |  | Lost wages for substitute labourers |  |  |  |  |  |  | |
|  |  | No substitute | Person-day | 980 | 0.0 | 0 | 0.00 | 0.00 | |
|  |  | 1 – 4 years old | Person-day | 0 | 0.0 | 0 | 0.00 | 0.00 | |
|  |  | 5 – 14 years old | Person-day | 8 | 3.0 | 24 | 0.00 | 0.00 | |
|  |  | >=15 years old | Person-day | 41 | 4.8 | 195 | 9.91 | 1,929.97 | |
|  |  |  |  |  |  |  |  |  | |
|  |  | Lost wages for companions |  |  |  |  |  |  | |
|  |  | No companion | Person-day | 166 | 0.0 | 0 | 0.00 | 0.00 | |
|  |  | 1 – 4 years old | Person-day | 0 | 0.0 | 0 | 0.00 | 0.00 | |
|  |  | 5 – 14 years old | Person-day | 8 | 1.8 | 14 | 0.00 | 0.00 | |
|  |  | >=15 years old | Person-day | 855 | 7.7 | 6609 | 9.91 | 65,496.68 | |
|  |  | **Total indirect costs** |  |  |  |  |  | **126,111.69** | |
| **Total costs by *P. vivax*** | | |  |  |  |  |  | **130,834.80** | |

| **Table S8. Direct and indirect costs from the patient perspective for uncomplicated malaria by *P.falciparum* in Mazan district 2017 (US$)** | | | | | | | | |
| --- | --- | --- | --- | --- | --- | --- | --- | --- |
|  |  |  |  |  |  |  |  |  |
| **Cost category** | | | Unit | Number of episodes | Average person-days lost | Quantity | Unit costs | Sub-total costs (US$) |
|  |  |  |  |  |  |  |  |  |
|  |  |  |  |  |  |  |  |  |
| ***P. falciparum*** *(n=315)* | | |  |  |  |  |  |  |
|  | **Direct cost** | |  |  |  |  |  |  |
|  |  | Medicaments | Per episode | 315 |  | 315 | 0.00 | 0.00 |
|  |  | Transport cost for patient | Round trip | 315 |  | 315 | 2.78 | 875.70 |
|  |  | Transport cost for companions | Round trip | 315 |  | 315 | 0.64 | 201.60 |
|  |  | Other cost | Per episode | 315 |  | 315 | 0.15 | 47.25 |
|  |  | **Total direct costs** |  |  |  |  |  | **1,124.55** |
|  |  |  |  |  |  |  |  |  |
|  | **Indirect costs** | |  |  |  |  |  |  |
|  |  | Lost wages for patient |  |  |  |  |  |  |
|  |  | 1 – 4 years old | Person-day | 29 | 5.0 | 145 | 0.00 | 0.00 |
|  |  | 5 – 14 years old | Person-day | 69 | 5.4 | 371 | 0.00 | 0.00 |
|  |  | >=15 years old | Person-day | 217 | 11.8 | 2561 | 9.91 | 25,375.55 |
|  |  |  |  |  |  |  |  |  |
|  |  | Lost wages for substitute labourers |  |  |  |  |  |  |
|  |  | No substitute | Person-day | 272 | 0.0 | 0 | 0.00 | 0.00 |
|  |  | 1 – 4 years old | Person-day | 0 | 0.0 | 0 | 0.00 | 0.00 |
|  |  | 5 – 14 years old | Person-day | 6 | 7.5 | 45 | 0.00 | 0.00 |
|  |  | >=15 years old | Person-day | 37 | 7.4 | 275 | 9.91 | 2,728.02 |
|  |  |  |  |  |  |  |  |  |
|  |  | Lost wages for companions |  |  |  |  |  |  |
|  |  | No companion | Person-day | 81 | 0.0 | 0 | 0.00 | 0.00 |
|  |  | 1 – 4 years old | Person-day | 0 | 0.0 | 0 | 0.00 | 0.00 |
|  |  | 5 – 14 years old | Person-day | 6 | 10.9 | 66 | 0.00 | 0.00 |
|  |  | >=15 years old | Person-day | 228 | 6.9 | 1562 | 9.91 | 15,477.44 |
|  |  | **Total indirect costs** |  |  |  |  |  | **43,581.01** |
|  |  |  |  |  |  |  |  |  |
| **Total costs by *P. falciparum*** | | |  |  |  |  |  | **44,705.56** |
